# Impact of access to improved water and sanitation on diarrhea reduction among rural under-five children in low and middle-income countries: a propensity score matched analysis

**DOI:** 10.1186/s41182-023-00525-9

**Published:** 2023-06-15

**Authors:** Mehari Woldemariam Merid, Adugnaw Zeleke Alem, Dagmawi Chilot, Daniel Gashaneh Belay, Anteneh Ayelign Kibret, Melaku Hunie Asratie, Yadelew Yimer Shibabaw, Fantu Mamo Aragaw

**Affiliations:** 1 Department of Epidemiology and Biostatistics, Institute of Public Health, College of Medicine and Health Sciences, University of Gondar, Gondar, Ethiopia; 2 Department of Human Physiology, School of Medicine, College of Medicine and Health Science, University of Gondar, Gondar, Ethiopia; 3 Center for Innovative Drug Development and Therapeutic Trials for Africa (CDT-Africa), College of Health Sciences, Addis Ababa University, Addis Ababa, Ethiopia; 4 Department of Human Anatomy, College of Medicine and Health Sciences, University of Gondar, Gondar, Ethiopia; 5 Department of Women’s and Family Health, School of Midwifery, College of Medicine and Health Sciences, University of Gondar, Gondar, Ethiopia; 6 Department of Biochemistry, School of Medicine, College of Medicine and Health Science, University of Gondar, Gondar, Ethiopia

**Keywords:** Improved water, Low and middle-income countries, Propensity score matching, Sanitation, Sustainable Development Goals

## Abstract

**Background:**

Diarrhea, the second leading cause of child morbidity and mortality worldwide, is responsible for more than 90% of deaths in children under 5 years of age in low and middle-income countries (LMICs). The high burden of diarrhea is mainly attributable to the limited access to improved water and sanitation. However, the impacts of improved sanitation and drinking water in preventing diarrheal diseases are not well understood. Therefore, this study estimated both the independent and joint effects of improved sanitation and water on diarrhea occurrence among rural under-five children in LMICs.

**Methods:**

The current study utilized secondary data from the Demographic and Health Survey (DHS) datasets conducted between 2016 and 2021 in 27 LMICs. A total weighted sample of 330,866 under-five children was included in the study. We employed propensity score matching analysis (PSMA) to examine the effects of accessing improved water and sanitation on childhood diarrheal disease reduction.

**Results:**

The prevalence of diarrhea among children under 5 years of age in rural LMICs was 11.02% (95% CI; 10.91%, 11.31%). The probability of developing diarrhea among under-five children from households with improved sanitation and water was 16.6% (Average Treatment Effect on the Treated (ATT) = − 0.166) and 7.4% (ATT = − 0.074) times less likely among those from households with unimproved sanitation and water, respectively. Access to improved water and sanitation is significantly associated with a 24.5% (ATT = − 0.245) reduction of diarrheal disease among under-five children.

**Conclusions:**

Improved sanitation and drinking water source reduced the risk of diarrhea among under-five children in LMIC. The effects of both interventions (improved water and sanitation) had a larger impact on the reduction of diarrheal disease than the improvements to water or sanitation alone. Therefore, achieving Sustainable Development Goal 6 (SDG 6) is key to reducing diarrhea among rural under-five children.

**Supplementary Information:**

The online version contains supplementary material available at 10.1186/s41182-023-00525-9.

## Background

Diarrhea is the passage of three or more loose or liquid stools per 24 h (or more frequent passage that is different from normal) [1]. Globally, 1.7 billion cases of diarrhea occur each year, killing more than 525,000 children under the age of five annually. This accounts for 8% of all causes of death in under-five children [2, 3]. Diarrhea is the second leading cause of child morbidity and mortality worldwide and is responsible for more than 90% of deaths in children under 5 years of age in low and middle-income countries (LMICs) [2].

An inequitable proportion of diarrheal morbidity and mortality occurs among under-five children in LMICs where access to health care, improved water and sanitation is limited [4, 5]. Unimproved water and sanitation are the leading cause of diarrhea, responsible for 72·1% and 56·4% of diarrhea mortality in children younger than 5 years, respectively [6].

Sustainable Development Goal 6 (SDG-6) aims to achieve universal and equitable access to drinking water, sanitation, and hygiene for all by 2030 [7]. However, in 2020, more than a quarter (26%) and nearly half (46%) of the world’s population lacked access to improved drinking water and sanitation, respectively. By 2030, billions of people will be without access to improved water and sanitation unless progress quadruples [8]. In addition, there are significant disparities in access to improved water and sanitation across regions and between urban and rural areas. Access to improved sanitation and drinking water is lower in rural areas than in urban areas. In particular, people without access to improved water and sanitation facilities are disproportionately concentrated in rural parts of Southern Asia and sub-Saharan Africa regions [9–12]. Also it has been noted that, in rural settings, only 2 out of 10 people have access to basic drinking water services [13].

The various socio-demographic, socio-economic, behavioral, and environmental factors are known to contribute to the occurrence of diarrhea among under-five children in the LMICs [14–24]. The association between drinking water source and sanitation and childhood diarrhea has been documented in the literatures [14, 18, 22, 24–29]. The provision of an improved drinking water source and sanitation reduced diarrhea risk by 52% and 24%, respectively, in LMICs [22]. However, most of the previous studies investigated the association between water source and sanitation and diarrhea used the conventional logistic regression model [14, 18, 22, 24–28]. Unlike the conventional logistic regression model, the use of propensity score matched analysis offers a better option compared to conventional logistic regression analyses in controlling for the confounding that may exist in analyzing associations between independent variables and the outcome variable.

Furthermore, the previous studies conducted in different countries showed that access to improved water and sanitation facilities is low in rural areas [30–33], however, there is a scarcity of information on effects of improved water and sanitation on reduction of diarrhea in rural settings. Therefore, understanding the causal effect of improved water and sanitation on the reduction of diarrhea in high-risk population will help public health practitioners to identify, implement and evaluate evidence-based specific interventions to tackle the burden of diarrheal diseases among under-five children in LMICs. Therefore, this study aimed to investigate effectiveness of access to improved sanitation and water on under-five diarrheal reduction using propensity score matching.

## Methods

### Study design and settings

The study was based on the national community-based cross-sectional survey data conducted between 2016 and 2021 in 27 LMICs. Low and middle-income countries are home to 62% of the global under-five population [34]. Generally, rural areas have poor health outcomes than urban areas in LMICs [35].

### Data source and study population

We used appended Demographic and Health Survey (DHS) datasets of 27 LMICs conducted during the SDG era. The DHS is a nationally representative survey, collected every five years in LMICs to provide up-to-date information on the background characteristics of all household members, neighborhood infrastructure, housing conditions, and access to basic services for policy development, planning, and evaluation of population and health programs in the respective countries. Data were collected by trained professionals and the questionnaire was conceptualized to each country’s context. Moreover, each country pre-tested questionnaire before actual data collection. Each country survey consists of different types of datasets such as household member recode (PR), individual (women’s) recode (IR), children’s recode (KR), births recode (BR), men’s recode (MR) and household recode (HR) datasets. For this study, we merged the household member recode (PR) and the Kids Record datasets (KR file). The dependent, treatment, and matching variables for each country were extracted from both datasets. In this analysis, a total weighted sample of 278,111 rural children aged 6–59 months was included.

### Study variables and measurement

#### Dependent variable

The outcome variable for this study was childhood diarrhea. Diarrhea was assessed by asking the mothers/caregivers “has your child had diarrhea in the last 2 weeks?” It was recoded as “one” if mothers/caregivers responded yes to the question and “zero” if mothers/caregivers answered no to the question.

#### Treatment variables

The treatment variables were household access to improved water and sanitation. Household access to water sources and sanitation facilities were categorized based on the World Health Organization (WHO) and United Nations International Children’s Emergency Fund (UNICEF) definitions as “improved” and “unimproved”. Accordingly, the source of water was categorized as “improved” if the source of water is from protected well, protected spring, rainwater, piped into dwelling, piped to yard/plot, public tap/standpipe, tube well or borehole, piped to neighbor or bottled water and “unimproved” if the sources of water is unprotected dug well, unprotected spring, surface water, vendor-provided water, bottled water, or tanker truck water. Households using ventilated improved pit latrine, pour-flush latrine, simple pit latrine, pit latrine with slab, composting toilet, or if toilet flushed to a public sewer or septic system were coded as “improved toilet” and households using toilet characterized by pit latrine—without slab, flushing to somewhere else, bucket toilet, traditional dry vault, dry toilet, or other toilet were coded as “unimproved toilet”.

#### Matching variables

On the basis of available literature, a number of household, maternal/caregiver, and child related variables were included as covariates. The lists of included variables were: maternal age, maternal educational level, household wealth status, frequency of listening to the radio, frequency of watching television, frequency of reading newspaper/magazines, hand washing, treating water, parity, family size, age of the child, birth order, stunting, child underweight, wasting, number of under-five children in the household and breastfeeding.

Treating water: Households reported the use of one or more of the following methods to treat drinking water prior to drinking and categorized as “yes” if they did so and “no” otherwise; boiling, adding bleach/chlorine, straining through a cloth, using a water filter, solar disinfection, letting it stand and settle were considered as yes/using appropriate water treatment.

##### Wasting

Child was categorized as wasted if weight for height (WAZ) is < − 2 standard deviation (SD) and normal WAZ/not wasted if WAZ ≥ − 2SD from the median of the WHO reference population.

##### Underweight

A child was considered to be underweight if their underweight for age is < − 2 SD from the median of the WHO reference population.

##### Stunting

Child was categorized as stunted if height for age (HAZ) is < − 2 SD and normal HAZ/not stunted if HAZ -2SD and above from the median of the WHO reference population.

### Data analysis

Descriptive analysis was first undertaken to estimate the prevalence of diarrhea among under-five children according to household access to water and sanitation. This study used PSM to draw causal inferences of household access to water and sanitation to childhood diarrhea. The analysis has been carried out in three separate models. In the first model, we compared “improved sanitation” with “unimproved sanitation”. In the second model we compared “access to improved water” with “unimproved water”. Finally, we compared “access to both improved water and sanitation” with “unimproved water and sanitation”. We generated the propensity score by using pscore Stata command.

The common support option was employed to limit testing of the balancing property to only treated mothers whose propensity score for diarrhea was included within the range of propensity scores. The average treatment effect on the treated (ATT) was estimated to assess the impact of treatment variables on outcome variable. A one-to-one nearest neighbor matching technique within a caliper range of ± 0.1 was performed. Balancing tests were evaluated graphically by density plot and statistically by using pstest Stata command.

## Results

### Background characteristics of respondents

The study included 278,111 children under the age of five. Of these, 113,582 (40.8%) were in the 0–11 months age group and 94,577 (36.5%) were stunted. More than half (54.7%) of the children were born to women of age 25–34 years, and 84,790 (30.5%) had no formal education. Nearly two-thirds (*n* = 183,236, 65.9%) of the households had access to an improved water source, and 158,695 (57.1) had improved toilet facilities. Nearly two-thirds (*n* = 182,430, 65.6%) of households treated water before drinking (Table 1).

**Table 1:** Background characteristics of respondents in LMICs

Variable	Frequency	Percent
Age of mother		
15–24	85,222	30.6
25–34	152,152	54.7
35–49	40,737	14.7
Age of child in months		
0–11	113,582	40.8
12–36	108,934	39.2
37–59	55,595	20.0
Maternal education		
Not educated	84,790	30.5
Primary	56,472	20.3
Secondary	114,499	41.2
Higher	22,350	8.0
Wealth status		
Poorest	90,728	32.6
Poorer	75,538	27.2
Middle	57,293	20.6
Richer	37,220	13.4
Richest	17,332	6.2
Handwashing facility		
No	108,772	45.3
Yes	131,501	54.7
Treating water		
No	182,430	65.6
Yes	95,643	34.4
Frequency of watching television		
Not at all	134,083	48.2
Less than once a week	52,174	18.8
At least once a week	91,313	32.8
Almost every day	522	0.2
Frequency of listening to radio		
Not at all	214,067	77.0
Less than once a week	34,422	12.4
At least once a week	27,667	9.9
Almost every day	1,931	0.7
Frequency of reading newspaper/magazine		
Not at all	226,062	81.3
Less than once a week	35,507	12.8
At least once a week	16,383	5.9
Almost every day	139	0.05
Parity		
Primiparous	58,991	21.2
Multiparous	188,882	67.9
Grand multiparous	30,238	10.9
Family size		
< 5	115,585	41.6
≥ 5	162,526	58.4
Source of drinking water		
Unimproved	94,875	34.1
Improved	183,236	65.9
Type of toilet		
Unimproved	119,416	42.9
Improved	158,695	57.1
Birth weight		
Low	66,607	24.1
Normal	209,385	75.9
Stunting		
No	164,571	63.5
Yes	94,577	36.5
Underweight		
No	218,016	85.2
Yes	38,004	14.8
Wasting		
No	189,996	71.9
Yes	74,200	28.1
Number of under-five children in household		
< 2	222,941	80.2
> 2	55,170	19.8
Breast feeding		
Still breastfeed	170,529	71.3
Ever breastfed	54,340	22.7
Never breastfed	14,291	6.0

### Prevalence of diarrhea among under-five children

The prevalence of diarrhea among children under 5 years of age in rural LMICs was 11.0% (95% CI; 10.9%, 11.3%). The pooled prevalence of diarrhea among children under 5 years of age in rural areas without improved toilet facilities was 12.5% (95% CI; 12.3%, 12.6%), while the prevalence of diarrhea among children under 5 years of age who used improved toilet facilities was 9.7% (95% CI; 9.1%, 11.2%). The country with the highest prevalence of diarrhea among under-five children was Burundi (22.7%), followed by Uganda (20.7%) and Haiti (20.6%). Whereas the lowest prevalence of diarrhea was observed in Maldives (4.4%) followed by Bangladesh (4.8%) (Table 2).

**Table 2:** Prevalence of diarrhea among under-five children in LMICs

Countries	Survey year	Prevalence of diarrhea (%)				
		Source of drinking water		Type of toilet		Total
		Improved	Unimproved	Improved	Unimproved	
Maldives	2016/17	4.1	4.4	2.8	4.4	4.4
Nepal	2016	6.6	7.4	6.3	9.5	7.4
Bangladesh	2017/18	4.8	5.0	4.9	4.7	4.8
India	2019/21	7.6	7.7	7.2	8.6	7.7
Pakistan	2017	18.9	19.2	18.1	19.6	19.2
Timor-Leste	2016	8.3	9.1	8.6	9.7	9.1
Albania	2017/18	6.9	8.2	7.8	13.4	8.2
Tajikistan	2017	13.3	13.4	14.4	14.2	13.4
Jordan	2017/18	9.7	9.8	9.7	11.5	9.8
Benin	2017/18	10.0	11.1	7.3	11.9	11.1
Cameroon	2018	11.1	11.5	11.2	12.3	11.5
Mauritania	2019/21	13.1	13.6	12.8	14.0	13.6
Gambia	2019/20	16.8	18.9	18.2	19.3	18.7
Guinea	2018	13.6	14.6	13.5	15.1	14.6
Liberia	2019/20	19.2	17.9	17.2	18.1	17.9
Mali	2018	16.6	17.9	16.4	19.2	17.9
Nigeria	2018	14.0	14.9	13.9	15.5	14.9
Sierra leone	2019	6.7	7.3	7.1	7.4	7.3
Burundi	2016/17	22.3	23.0	21.7	24.1	23.0
Ethiopia	2016	12.0	12.3	10.3	12.1	12.0
Madagascar	2021	8.8	9.1	8.7	9.2	9.1
Rwanda	2019/20	14.9	14.3	14.9	16.1	14.9
South Africa	2016	11.6	13.2	13.2	13.2	13.2
Uganda	2016	20.8	20.7	20.6	20.7	20.7
Haiti	2016/17	18.6	20.6	18.5	21.7	20.6
Zambia	2018	14.1	15.6	15.7	15.6	15.6
Papua New Guinea	2016–18	12.4	14.5	12.6	15.1	14.5
Overall		11.1	12.0	9.7	12.5	11.0

### Description of the estimated propensity scores

A comparison of improved vs unimproved sanitation showed that the mean propensity score was 0.595 with a standard deviation (SD) of ± 0.225. The region of common support between the treated and the control group ranged from 0.104 to 0.991 on the propensity score. The overall mean propensity score was 0.372 (SD ± 0.09) for improved and unimproved water. The region of common support between the improved (treated) and the control (unimproved) water ranged from 0.152 to 0.738. For both improved water and sanitation, the overall mean propensity score was 0.201 (SD ± 0.08). The region of common support between treated and control ranged from 0.06 to 0.647.

**Fig. 1:**
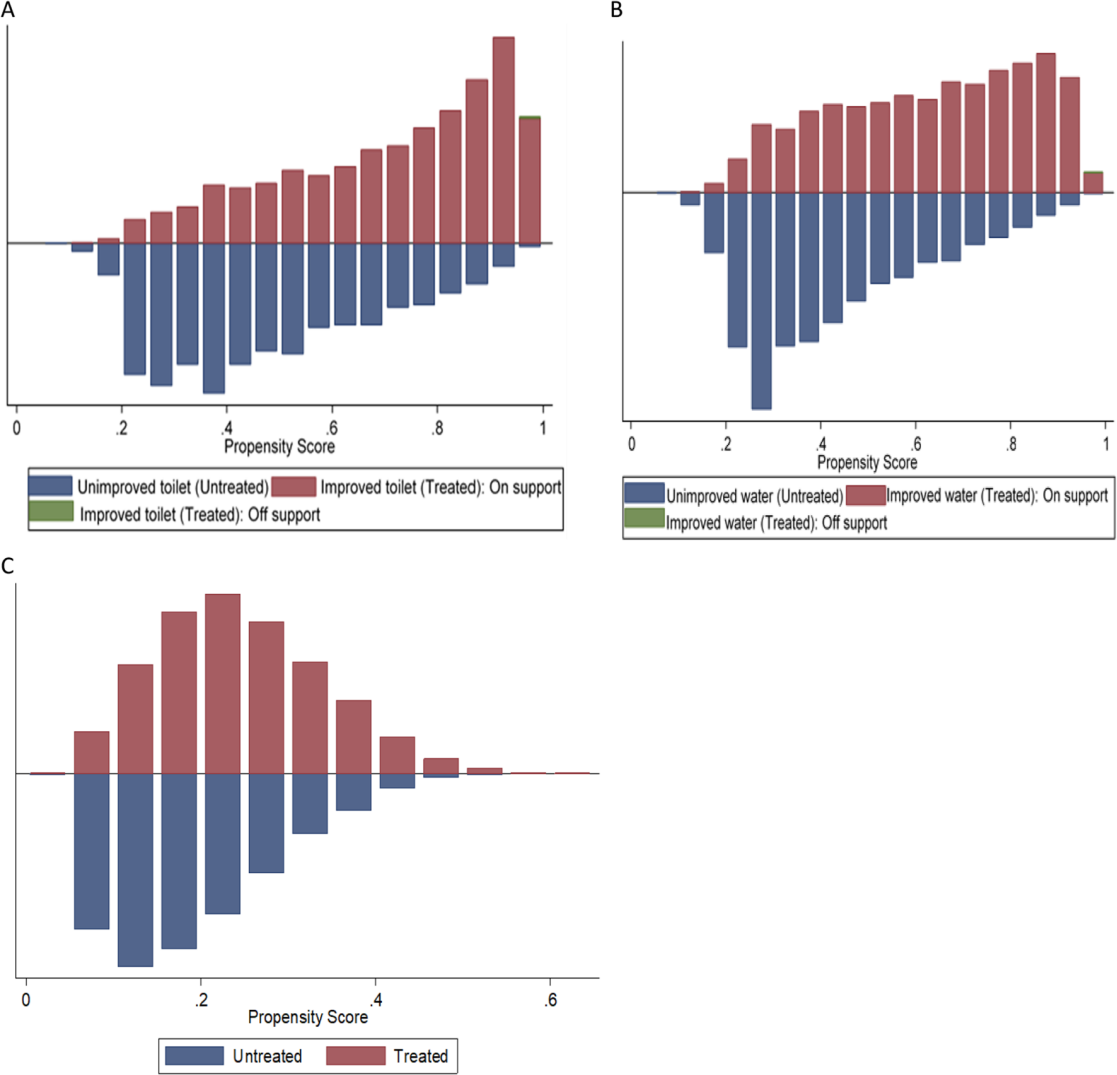
Histogram of propensity score distribution for treated and control children: **A** for type of toilet facility, **B** for source of drinking water and **C** for improved water and sanitation

Figure 1A–C shows the balance of the propensity score distributions between the treatment and control groups. The figures demonstrate adequate overlap in the propensity score distributions between treated and control groups. Figure 1A indicates that when comparing improved with unimproved sanitation, a total of 66 treated observations out of 256,020 observations were discarded due to common support. Figure 1B shows that when comparing improved with unimproved water, a total of 42 treated observations out of 256,020 observations were discarded due to common support.

### Impact assessment

The matched analysis showed that the probability of developing diarrhea was 7.4% (ATT = − 0.074) lower among children from households with improved sanitation compared with children from households with unimproved sanitation. Children in households with improved drinking water were 16.6% less likely to develop diarrhea than children in households with unimproved drinking water (ATT = − 0.166). Meanwhile, having both improved water and sanitation are significantly associated 24.5% (ATT = − 0.245) reduction of diarrheal disease among under-five children (Table 3).

**Table 3:** Unmatched and matched estimates of treatment variables on occurrence of diarrhea among under-five children in LMICs

Variable	Sample	Treated	Control	Difference	SE	95% CI
Improved water	Unmatched	0.088	0.112	− 0.024	0.001	
	ATT	0.088	0.162	− 0.074	0.003	− 0.082 to − 0.071
Improved sanitation	Unmatched	0.101	0.113	− 0.012	0.001	
	ATT	0.101	0.267	− 0.166	0.002	− 0.169 to − 0.161
Both improved water and sanitation	Unmatched	0.089	0.223	− 0.134	0.002	
	ATT	0.089	0.334	− 0.245	0.003	− 0.251 to − 0.240

*ATT* average treatment effect on the treated, *CI* confidence interval

### Balancing test

**Fig. 2:**
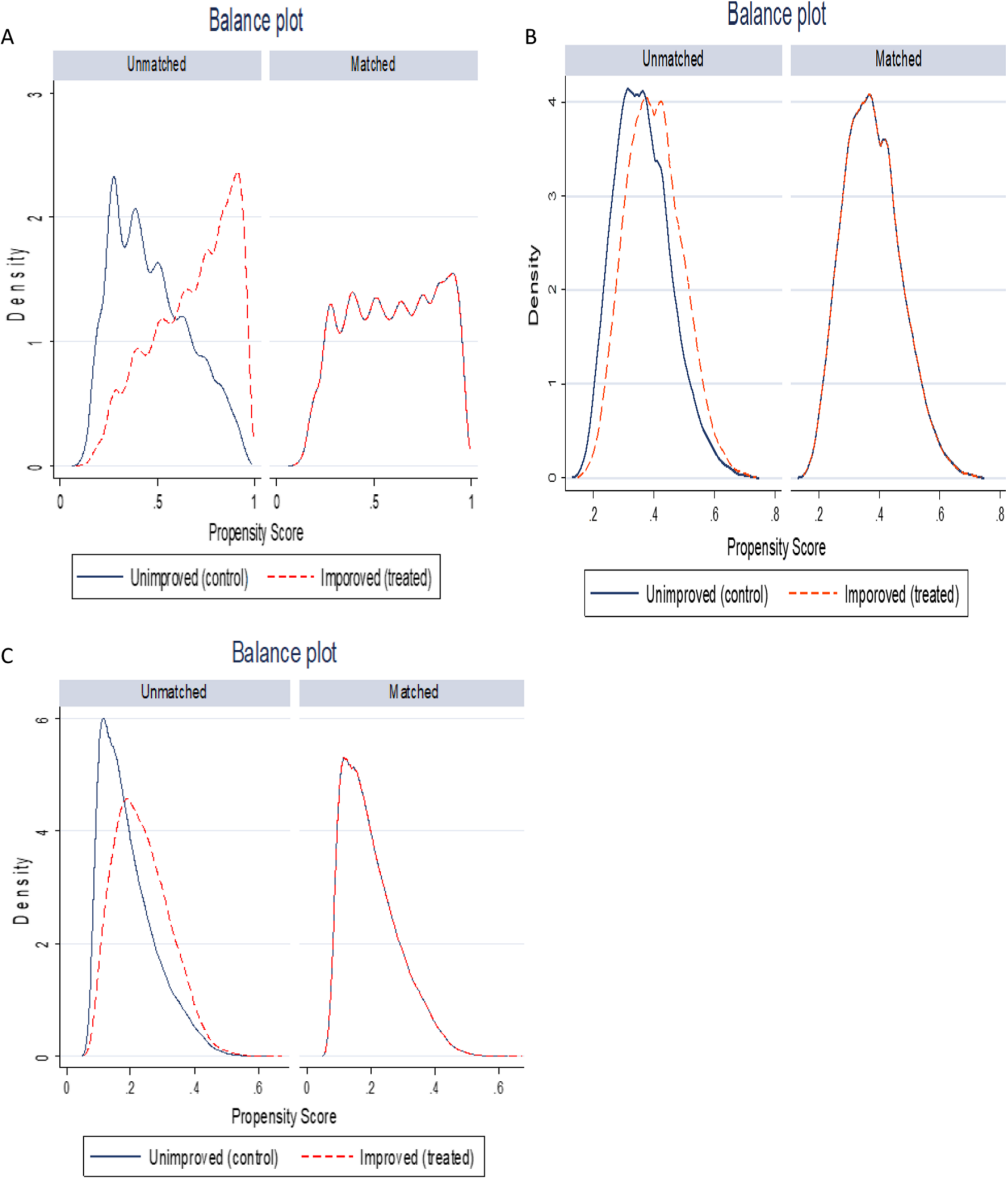
Kernel density plot of estimated propensity scores before and after matching: A for type of toilet facility, B for source of drinking water and C for improved water and sanitation

Figure 2A–C shows the distributions of the propensity scores before and after matching for treatment variables. Results indicate that the distributions of the propensity scores perfectly overlapped after matching for all treatment variables. This indicates covariates were sufficiently balanced after matching. Similarly, for almost all variables, the hypothesis of each variable is the same in the treated children and untreated children after matching was satisfied (*p*-value ≥ 0.05) (Additional file 1: Table S1)

## Discussion

This study is a large-scale study specifically intended to estimate the effect of access to improved sanitation and water on diarrhea reduction among under-five children in 27 LMICs. Although considerable progress has been made in reducing under-five mortality from 9.92 million (75 per 1000 live births) to 5 million (37 per 1000 live births) between 2000 and 2020, it remains a major public health issue signaling much remains to do. With the current progress, it is far more difficult to achieve the SDGs under-five mortality target of 25 deaths per 1000 live births by 2030 in most LMICs [36, 37]. Diarrhea is one of the major contributors to under-five children mortality in LMICs. More than three-fourths (78%) of childhood diarrheal deaths occurred in South Asia and sub-Saharan Africa [6]. This study used PSM to estimate effectiveness of access to improved sanitation and water on diarrhea reduction among under-five children in LMICs during the SDG era. The propensity score matching is a statistical method that attempts to estimate treatment effects with observational data. It offers an alternative approach for estimating treatment effects, policy, and program evaluation in cases where randomized controlled trials (RCT) are impossible or inappropriate [38, 39]. Using observational data, the PSM analysis in the present study showed that access to improved sanitation and drinking water significantly reduced the risk of diarrhea among under-five children.

This study revealed that improved sanitation was associated with a 16% reduction in the risk of diarrhea occurrence among under-five children after matching control and treated children. This finding is in agreement with other studies conducted elsewhere [17, 18, 21, 22, 24, 27, 28, 40]. A systematic and meta-analysis conducted by Wolf J et al. found that improved sanitation interventions can reduce the occurrence of diarrheal diseases by 24% among children in LMICs [22]. A study conducted in rural Ethiopia reported that children from households who had no access to toilet facilities were 1.50 to 4.8 times more likely to having of diarrhea compared to children from households who had access to toilet facilities [18, 24, 27, 40]. Also, a study conducted in rural Tanzania revealed that improved waste management reduced diarrhea risk by 63% among children [28]. However, previous studies were largely observational and used conventional regression models to investigate the association between access to improved sanitation and diarrhea. To our knowledge, this is the first study in LMICs to use PSM to estimate the effectiveness of access to improved sanitation and water on diarrhea reduction. Improving access to Water, Sanitation, and Hygiene (WASH) is a key intervention to improve child health and well-being by preventing the spread of communicable diseases. Poor WASH is the main cause of feco-oral transmitted infections, including diarrheal disease, which remains the major global public health problem. Besides, diarrheal diseases, poor WASH also contributes to increased risk of malaria, polio, and neglected tropical diseases (NTDs) such as trachoma, guinea worm, schistosomiasis, and helminths that have a debilitating effect on children’s health [41–43]. Improving WASH supports the achievement of many SDGs. It contributes to the achievement of improving child health (SDG 3), reducing malnutrition (SDG 2), ending child poverty (SDG 1), creating decent working conditions (SDG 8), and environmental protection and climate change (SDG 13) [41]. Despite, ensuring universal and equitable access to improved drinking water, sanitation and hygiene for all is one of the SDGs of the United Nations, its coverage remains low in LMICs [9–12].

Similarly, we observed a negative association between access to improved water and diarrhea occurrence among under-five children. This finding is consistent with a study conducted in LMICs which reported that the provision of improved drinking water reduced diarrhea risk by 52% [22]. Similarly, Ko SH et al. found that under-five children in households with drinking untreated are less likely to develop diarrhea in the rural areas of Myanmar [44]. This might be due to the fact that unimproved source of drinking water may carry pathogens that cause diarrhea.

Our finding also noted that access to both improved sanitation and water had a greater impact on the reduction of diarrhea. As such, we found that nearly a quarter (24.5%) of diarrhea cases among under-five children could be reduced by accessing improved sanitation and water in LMICs. So far, very limited studies have investigated the joint effects of improved water and sanitation services in preventing diarrheal disease. Consistent with our finding, Fuller et al. reported that both interventions had a larger impact on the reduction of diarrheal disease than the improvements to water or sanitation alone [45]. In fact, improved water sources had no meaningful effect on health if a community had unimproved sanitation since files can contaminate water sources. In contrast, a systematic and meta-analysis conducted in less developed countries revealed that multiple interventions (consisting of combined water, sanitation, and hygiene measures) were not more effective than interventions with a single focus (water, sanitation, or hygiene) [46]. A possible explanation for this difference could be in that our analysis included an up-to-date national representative data from the DHS. Second, we estimated using observational data by PSM approach instead of meta-analyses of intervention trials and observational studies as previous studies had done.

The current study has several strengths: To the best of our knowledge, this is the first study that has investigated both the independent and joint effects of improved water and sanitation on the occurrence of diarrhea among under-five children in LMICs. Using the large nationally representative sample, and PSM that reduces selection bias and confounding effect are also strengths of this study. Notwithstanding these strengths, our study has some limitations. First, PSM matches the treated with controls which leads to better estimates of treatment effect, however, estimates rely on the un confoundedness assumption. Therefore, bias due to unmeasured covariates is not accounted for leading to overestimated effects of improved water and sanitation on diarrhea. Second, even though the effect of improved water and sanitation may vary from setting to setting, country-specific estimates are not reported in this study.

## Conclusion

Improved sanitation and drinking water source reduced the risk of diarrhea significantly among under-five children in LMIC. The effects of both interventions (improved water and sanitation) had a larger impact on the reduction of diarrheal disease than the improvements to water or sanitation alone. Achieving SDG 6 is key in reducing diarrhea among rural under-five children. Therefore, policymakers should strengthen capacity and systems to enable all stakeholders to contribute effectively in order to scale-up access to improved water and sanitation in rural populations.

## Supplementary Information

**Additional file 1: Table S1.** Covariate balance check and absolute bias reduction

## Data Availability

Data are available online in a public, open-access repository (www.measuredhs.com/data).

## Abbreviations

ATT: Average treatment effect on the treated
DHS: Demographic and health survey
LMICs: Low and middle-income countries
PSM: Propensity score matching
WHO: World Health Organization
SDG: Sustainable Development Goal
