# Supplementary material for: Impact of access to improved water and sanitation on diarrhea reduction among rural under-five children in low and middle-income countries: a propensity score matched analysis
Source: Trop Med Health. 2023 Jun 15;51:36. doi: 10.1186/s41182-023-00525-9 (PMC10268525; doi:10.1186/s41182-023-00525-9)
Supplement: Supplementary file 1 — Additional file 1: Table S1. Covariate balance check and absolute bias reduction [file 41182_2023_525_MOESM1_ESM.docx]

**Additional file 1: Table S1**:- Covariate balance check and absolute bias reduction

| Variable | Sample | Treated | Control | %Bias | % reduction bias | t | p>t |
| --- | --- | --- | --- | --- | --- | --- | --- |
| Age of mother |  |  |  |  |  |  |  |
| 25-34 | Unmatched | 0.5731 | 0.5120 | 12.3 |  | 26.01 | <0.001 |
|  | Matched | 0.5735 | 0.5740 | -0.1 | 99.2 | -0.24 | 0.813 |
| 35-49 | Unmatched | 0.1359 | 0.1631 | -7.6 |  | -16.23 | <0.001 |
|  | Matched | 0.1354 | 0.1348 | 0.2 | 97.7 | 0.42 | 0.672 |
| Age of child in years |  |  |  |  |  |  |  |
| 2-3 | Unmatched | 0.3824 | 0.3707 | 2.4 |  | 5.11 | <0.001 |
|  | Matched | 0.3824 | 0.3837 | -0.3 | 89.0 | -0.62 | 0.534 |
| 4-5 | Unmatched | 0.1640 | 0.1587 | 1.4 |  | 3.04 | 0.002 |
|  | Matched | 0.1637 | 0.1654 | -0.5 | 68.5 | -1.06 | 0.291 |
| Maternal education |  |  |  |  |  |  |  |
| Primary | Unmatched | 0.1623 | 0.2408 | -19.7 |  | -42.17 | <0.001 |
|  | Matched | 0.1623 | 0.1564 | 1.5 | 92.4 | 3.82 | 0.234 |
| Secondary | Unmatched | 0.5174 | 0.3082 | 43.5 |  | 91.21 | <0.001 |
|  | Matched | 0.5173 | 0.5405 | -4.8 | 88.9 | -10.93 | 0.056 |
| Higher | Unmatched | 0.1198 | 0.0382 | 30.6 |  | 61.84 | <0.001 |
|  | Matched | 0.1196 | 0.1099 | 3.7 | 88.0 | 7.18 | 0.115 |
| Wealth status |  |  |  |  |  |  |  |
| Poorer | Unmatched | 0.2559 | 0.2876 | -7.1 |  | -15.13 | <0.001 |
|  | Matched | 0.2560 | 0.2679 | -2.7 | 62.8 | -6.30 | 0.279 |
| Middle | Unmatched | 0.2502 | 0.1596 | 22.6 |  | 47.05 | <0.001 |
|  | Matched | 0.2504 | 0.2458 | 1.1 | 94.9 | 2.50 | 0.012 |
| Richer | Unmatched | 0.1983 | 0.0657 | 39.9 |  | 81.07 | <0.001 |
|  | Matched | 0.1984 | 0.182 | 5.0 | 87.6 | 9.84 | 0.078 |
| Richest | Unmatched | 0.1057 | 0.0177 | 37.2 |  | 73.98 | <0.001 |
|  | Matched | 0.1052 | 0.1131 | -3.3 | 91.1 | -5.90 | 0.177 |
| Handwashing |  |  |  |  |  |  |  |
| Yes | Unmatched | 0.6478 | 0.3562 | 61.0 |  | 128.94 | <0.001 |
|  | Matched | 0.6476 | 0.6494 | -0.4 | 99.4 | -0.87 | 0.387 |
| Treating water |  |  |  |  |  |  |  |
| Yes | Unmatched | 0.4327 | 0.2733 | 33.8 |  | 70.79 | <0.001 |
|  | Matched | 0.4324 | 0.4447 | -2.6 | 92.3 | -5.84 | 0.345 |
| Frequency of watching television | |  |  |  |  |  |  |
| Less than once a week | Unmatched | 0.2123 | 0.1692 | 11.0 |  | 23.05 | <0.001 |
|  | Matched | 0.2124 | 0.1990 | 3.4 | 69.0 | 7.77 | 0.120 |
| At least once a week | Unmatched | 0.4447 | 0.2019 | 53.8 |  | 111.42 | <0.001 |
|  | Matched | 0.4444 | 0.4518 | -1.6 | 97.0 | -3.47 | 0.004 |
| Almost every day | Unmatched | 0.0020 | 0.0014 | 1.6 |  | 3.23 | 0.001 |
|  | Matched | 0.0020 | 0.0021 | -0.2 | 88.6 | -0.38 | 0.706 |
| Frequency of listening to radio | |  |  |  |  |  |  |
| Less than once a week | Unmatched | 0.1247 | 0.1174 | 2.2 |  | -1.94 | <0.001 |
|  | Matched | 0.1247 | 0.1278 | -0.9 | 59.1 | 5.33 | 0.053 |
| At least once a week | Unmatched | 0.0921 | 0.1173 | -8.3 |  | -17.65 | <0.001 |
|  | Matched | 0.0921 | 0.0864 | 1.9 | 77.5 | 4.69 | 0.412 |
| Almost every day | Unmatched | 0.0051 | 0.0094 | -5.1 |  | -11.05 | <0.001 |
|  | Matched | 0.0051 | 0.0046 | 0.6 | 89.1 | 1.59 | 0.113 |
| Frequency of reading newspaper/magazine | |  |  |  |  |  |  |
| Less than once a week | Unmatched | 0.1716 | 0.0859 | 25.8 |  | 53.15 | <0.001 |
|  | Matched | 0.1717 | 0.1645 | 2.2 | 91.6 | 4.50 | 0.178 |
| At least once a week | Unmatched | 0.0864 | 0.0296 | 24.5 |  | 49.63 | <0.001 |
|  | Matched | 0.0859 | 0.0875 | -0.7 | 97.2 | -1.33 | 0.183 |
| Almost every day | Unmatched | 0.0004 | 0.0007 | -1.1 |  | -2.43 | 0.015 |
|  | Matched | 0.0004 | 0.0003 | 0.7 | 41.4 | 1.96 | 0.051 |
| Parity |  |  |  |  |  |  |  |
| Multiparous | Unmatched | 0.6475 | 0.6423 | 1.1 |  | 2.28 | 0.023 |
|  | Matched | 0.6474 | 0.6470 | 0.1 | 92.8 | 0.18 | 0.855 |
| Grand Multiparous | Unmatched | 0.0674 | 0.1434 | -25.0 |  | -54.46 | <0.001 |
|  | Matched | 0.0674 | 0.0617 | 1.9 | 92.5 | 5.45 | 0.329 |
| Family size |  |  |  |  |  |  |  |
| ≥5 | Unmatched | 0.5641 | .5761 | -2.4 |  | -5.12 | <0.001 |
|  | Matched | 0.5642 | .56575 | -0.3 | 87.2 | -0.73 | 0.468 |
| Source of drinking water | |  |  |  |  |  |  |
| Improved | Unmatched | 0.3382 | 0.4228 | -17.5 |  | -37.17 | <0.001 |
|  | Matched | 0.3383 | 0.3761 | -7.8 | 55.4 | -18.52 | 0.092 |
| Birth weight |  |  |  |  |  |  |  |
| Normal | Unmatched | 0.7386 | 0.7817 | -10.1 |  | -21.23 | <0.001 |
|  | Matched | 0.7386 | 0.7366 | 0.5 | 95.4 | 1.07 | 0.285 |
| Stunting |  |  |  |  |  |  |  |
| Yes | Unmatched | 0.6694 | 0.6025 | 13.9 |  | 29.57 | <0.001 |
|  | Matched | 0.6693 | 0.6729 | -0.8 | 94.6 | -1.80 | 0.071 |
| Underweight |  |  |  |  |  |  |  |
| Yes | Unmatched | 0.8433 | 0.8450 | -0.9 |  | -0.98 | 0.038 |
|  | Matched | 0.8433 | 0.8465 | --0.5 | 90.1 | -2.08 | 0.327 |
| Wasting |  |  |  |  |  |  |  |
| Yes | Unmatched | 0.7545 | 0.7089 | 10.3 |  | 21.91 | <0.001 |
|  | Matched | 0.7544 | 0.7563 | -0.4 | 95.8 | -1.05 | 0.291 |
| Number of under five children in household | |  |  |  |  |  |  |
| >2 | Unmatched | 0.1528 | 0.1928 | -10.6 |  | -22.60 | <0.001 |
|  | Matched | 0.1529 | 0.1328 | 5.3 | 49.7 | 13.52 | 0.123 |
| Breast feeding |  |  |  |  |  |  |  |
| Ever breastfed | Unmatched | 0.1406 | 0.2574 | -29.6 |  | -63.90 | <0.001 |
|  | Matched | 0.1407 | 0.1228 | 4.5 | 84.7 | 12.40 | 0.063 |
| Never breastfed | Unmatched | 0.0648 | 0.0557 | 3.8 |  | 8.07 | <0.001 |
|  | Matched | 0.0648 | 0.0664 | -0.7 | 82.6 | -1.50 | 0.133 |
